# Around-Body Versus On-Body Motion Sensing: A Comparison of Efficacy Across a Range of Body Movements and Scales

**DOI:** 10.3390/bioengineering11111163

**Published:** 2024-11-19

**Authors:** Katelyn Rohrer, Luis De Anda, Camila Grubb, Zachary Hansen, Jordan Rodriguez, Greyson St Pierre, Sara Sheikhlary, Suleyman Omer, Binh Tran, Mehrail Lawendy, Farah Alqaraghuli, Chris Hedgecoke, Youssif Abdelkeder, Rebecca C. Slepian, Ethan Ross, Ryan Chung, Marvin J. Slepian

**Affiliations:** 1 Arizona Center for Accelerated Biomedical Innovation, University of Arizona, Tucson, AZ 85724, USA; katelynrohrer@arizona.edu (K.R.); lad44@arizona.edu (L.D.A.); cdgrubb@arizona.edu (C.G.); zacharysehansen@arizona.edu (Z.H.); jerodriguez@arizona.edu (J.R.); sheikhlary@arizona.edu (S.S.); beccaslepian@arizona.edu (R.C.S.); 2 Department of Computer Science, College of Science, University of Arizona, Tucson, AZ 85721, USA; 3 Department of Chemical Engineering, College of Engineering, University of Arizona, Tucson, AZ 85721, USA; gstpierre@arizona.edu (G.S.P.); farahalqaraghuli@arizona.edu (F.A.); 4 Department of Biomedical Engineering, College of Engineering, University of Arizona, Tucson, AZ 85721, USA; somer4@arizona.edu (S.O.); mlawendy@arizona.edu (M.L.); chedgecoke@arizona.edu (C.H.); youssifwafa@email.arizona.edu (Y.A.); 5 Department of Cellular and Molecular Medicine, College of Medicine, University of Arizona, Tucson, AZ 85721, USA; bttran@arizona.edu; 6 Department of Medicine, Sarver Heart Center, University of Arizona, Tucson, AZ 85721, USA; ecross1@arizona.edu (E.R.); chungr@arizona.edu (R.C.)

**Keywords:** motion capture, motion analysis, on-body sensing, around-body sensing, wearable sensors, stretchable electronics, marker tracking, human locomotion

## Abstract

Motion is vital for life. Currently, the clinical assessment of motion abnormalities is largely qualitative. We previously developed methods to quantitatively assess motion using visual detection systems (around-body) and stretchable electronic sensors (on-body). Here we compare the efficacy of these methods across predefined motions, hypothesizing that the around-body system detects motion with similar accuracy as on-body sensors. Six human volunteers performed six defined motions covering three excursion lengths, small, medium, and large, which were analyzed via both around-body visual marker detection (MoCa version 1.0) and on-body stretchable electronic sensors (BioStamp version 1.0). Data from each system was compared as to the extent of trackability and comparative efficacy between systems. Both systems successfully detected motions, allowing quantitative analysis. Angular displacement between systems had the highest agreement efficiency for the bicep curl and body lean motion, with 73.24% and 65.35%, respectively. The finger pinch motion had an agreement efficiency of 36.71% and chest abduction/adduction had 45.55%. Shoulder abduction/adduction and shoulder flexion/extension motions had the lowest agreement efficiencies with 24.49% and 26.28%, respectively. MoCa was comparable to BioStamp in terms of angular displacement, though velocity and linear speed output could benefit from additional processing. Our findings demonstrate comparable efficacy for non-contact motion detection to that of on-body sensor detection, and offers insight as to the best system selection for specific clinical uses based on the use-case of the desired motion being analyzed.

## 1. Introduction

Human motion is vital for life. In a wide variety of disease states, mobility is often compromised, either due to direct pathology involving neuromuscular and skeletal function, or indirectly via systemic functional, biochemical, or inflammatory alterations [1,2,3,4,5,6]. To aid in overall disease management, motion analysis has emerged as a potential diagnostic tool to guide both direct mobility rehabilitation as well as systemic therapies impacting mobility [7,8,9]. Great strides have been made in recent years in improving both the accessibility and usability of motion analysis systems, as well as in defining optimal specific use applications [10,11,12]. In recent years there has been a transition from strict hospital and facility-based motion labs to a wide range of portable and wearable technologies being employed [13,14]. Based on this technological evolution, motion analysis today may be broadly categorized as either “around-body”, i.e., via non-contact observation/detection systems, or “on-body”, via a range of wearable sensors detecting attached subject motion [14,15]. Our group has been involved in the development and testing of both of these types of systems, including on-body sensors utilizing stretchable electronics containing accelerometers and gyroscopes [16] as well as around-body systems utilizing visual marker detection and computational motion tracking [17]. It has become clear from this work, and the work of others, that each form of motion detection has functional nuanced differences, as to the ability to detect motion at a range of distances and involving differing geometric scales, with specific characteristics potentially favoring particular situational use [18,19,20,21].

The BioStamp (MC 10, Lexington, MA, USA) is an example of a portable, small-footprint on-body motion sensor, well suited for out-of-hospital home use, developed and studied in collaboration with our group [22]. The BioStamp is a wearable, conformal patch fabricated with pliant materials and containing stretchable electronics, capable of measuring motion with six degrees of freedom [22]. The BioStamp contains embedded accelerometers and gyroscopes, able to record acceleration and angular velocity [22]. In previous work we described details of this sensor system and demonstrated its ability to capture motion, as well as define a “motion envelope” for a given limb movement [22]. In other work we demonstrated the ability of the BioStamp to delineate signatures of specific motion tasks [23]. In general, the BioStamp provides detailed motion information, appearing to function best for the capture and quantitation of motion for the specific focal body region to which it is applied.

In contrast, the Mobile Motion Capture Analysis (MoCa) is an example of a low cost, video-based around-body motion tracking system able to detect and quantitate movement in a non-contact fashion [17,24]. MoCa uses brightly colored inexpensive markers (<=10 mm^2^ in size) and a smart-phone video camera to track positional movement. In prior work, we described this system and defined its operating parameters and boundaries in terms of distance tracked, marker identification and discrimination, and the effect of illumination and speed on motion detection [17]. Overall, MoCa appears well suited for the capture and quantitation of large region motions.

While both of these systems offer great advantages for out-of-hospital motion detection, what remains unknown is their comparative efficacy over a range of motions. In an effort to guide operational choice and the translation of these systems for the best situational and clinical use it is vital to define how these systems compare as to the detection of movements of differing size body components, e.g., whole body vs. limbs vs. digits. Over what size region is each best suited for motion detection? How well is each suited to detect, capture, and quantitate angular displacement, angular velocity, and linear speed? Over what speed of motion? In this present study, we conducted a comparative study to define and clarify these differences. We hypothesized that around-body motion detection with the MoCa system will provide similar data to that of the wearable on-body BioStamp system, though with identifiable differences in specific use cases and conditions. As a first step, we examined the ability of each system to detect and track specific, defined identical movements. Next, we examined the ability of each system to quantitate motion across differing movement excursions or “sizes”, herein defined in terms of dimensional areas, i.e., as small (<1 sq ft), medium (1–5 sq ft), and large (6–10 sq ft). We then compared the efficacy of each system across motion endpoint variables for each of these movement sizes, with efficacy defined in terms of angular displacement, angular velocity, and linear speed. We then examined the effect of the speed of movement on the comparative efficacy of each system. Finally, we discuss our findings in the context of translatable clinical utility as to which each type of system is best.

In summary, the contributions of this work are as follows: MoCa, a novel computer-vision “around-body” motion analysis system is compared to BioStamp, an established gyroscopic and accelerometer IMU. Meaningful angular and linear data are extracted out of both systems and compared against one another. Their statistical results are analyzed in the context of the medical domain, and next steps are outlined for the applicability of either system in a clinical application.

## 2. Materials and Methods

### 2.1. Materials

Six volunteers were enrolled in this study, 3 male, 3 female, ages 15–30, all providing informed consent, under study approval by UA IRB 2107032177.

Around-body testing via MoCa: General materials included neon pink or green construction paper, a ruler and tape measures, a metronome, and Apple iPhone 8, XR, or newer models. Pink, orange, and green markers were used as identification points. Markers were cut from construction paper as 2 mm squares for small movements and 10 mm squares for medium and large movements (Table 1). A low-stick adhesive was affixed to the back of each marker to allow direct skin adhesion, or adhesion on top of an affixed BioStamp. A smartphone camera with 1080 p resolution, recording at 60 frames per second, was used to track each movement, ensuring that the video produced was of high quality to accurately record the motion. The phone was supported by a variable-length tripod.

On-body testing via BioStamps: The BioStampRC^®^ (Model No. BRCS01) and kit (charging station for stamps, adhesive strips, recording tablet (Samsung Galaxy Tab. A), and conductive gel), were obtained from MC10, Inc. (Lexington, MA, USA). The BioStamp is a thin, pliable device containing stretchable electronics directly applied to the skin surface (3.4 cm × 6.6 cm × 0.45 cm; weight = 7 g). The BioStamp is controlled via an embedded microcontroller for recording bio-signals and the transmission of data via WiFi to the MC10 Investigator Portal or broadcasting wirelessly via Bluetooth to the MC10 Discovery app, pre-loaded on an Android™ tablet. Prior to the application of BioStamp to a subject, the sensor was configured to select the measurement modality (3 axis accelerometer, 3 axis gyroscope, ECG, EMG, or combination), sampling frequency (50–250 Hz), and measurement range (±2–16 G for accel.; ±250–4000°/s for gyro). Once configured, the BioStamp was applied to the subject and was selected to start or stop recording and sync data from the tablet. Data were then uploaded to the cloud where they could be accessed and downloaded from the MC10 Investigator Portal website. Participants were supported by a table and/or chair to perform recorded movements.

As to metrics, the BioStamp recorded in 3 dimensions and 62.5 Hz whereas MoCa recorded in 2 dimensions and the camera recording settings were set to 60 frames per second in HD (1080 × 1920 pixels) quality.

At most, five BioStamps were used to measure movements. The BioStamps were controlled by a tablet and cell phone combination from the BioStamps kit. The combination links to a docking stand that synchronized the in-use BioStamps, as well as uploaded data from the BioStamps to the associated website, MC10. The BioStamps were attached to the subject with surgical tape and medical wrap.

### 2.2. Experimental Methods

Subjects performed 6 standard movements in 30 s increments at slow and fast paces. Each movement was repeated three times at each speed for a total of six runs per standard movement per subject. These movements were simultaneously recorded with the MoCa and BioStamp systems. The six standard movements were categorized based on area requirements: (1) small movements (<1 sq ft)—finger pinch, (2) medium movements (1–5 sq ft)—bicep curl, chest abduction/adduction, shoulder abduction/adduction, and shoulder flexion/extension, and (3) large movements (>5 sq ft)—body lean (Table 2).

### 2.3. Generic Protocol

**Figure 1:**
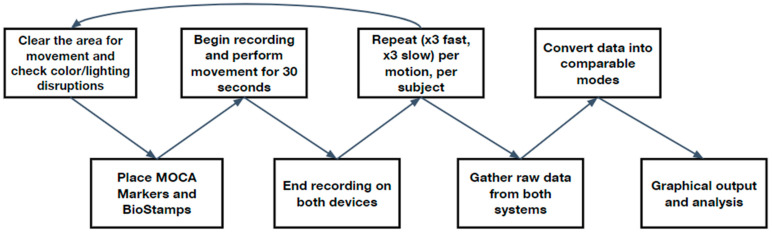
Outlined steps for recording the runs for both MoCa and BioStamp. The procedure covers the setting up of the area for recording the data transformation into graphs.

To compare the efficacy of MoCa to BioStamp, the protocol outlined in Figure 1 was employed; each trial recorded MoCa and BioStamp data simultaneously. Once repeated, the raw data from both systems were processed and compared.

To set up the MoCa system, two cameras were placed at the same height as the pivot marker and parallel to the subject. The primary tracking camera was placed perpendicular to the primary axis of movement for the test motion, and the secondary camera was placed directly to the left or right of the subject to ensure that the third axis of motion remained stable. Next, the identification markers were set up by placing different color markers on pivot, endpoint, and intermediate locations as per the directions for each motion. These markers were required to be directly facing the primary camera and remained primarily visible to the camera in order to consistently record axis movement. Once both systems were set up, the subject performed the movement for 30 s at both fast and slow speeds (Table 1). The speed of movement was controlled by the time per repetition (Table 1). At the end of the movement, the subject returned to the starting position and waited for all motion capture devices to stop recording.

For the BioStamp system, sensors were placed based on the subject at defined reproducible locations. BioStamps were secured bilaterally with medical tape and subsequently wrapped in gauze to ensure no part of the stamp would move or detach from the subject’s skin during the study.

#### 2.3.1. Finger Pinch (FingerP) Movement Protocol

**Figure 2:**
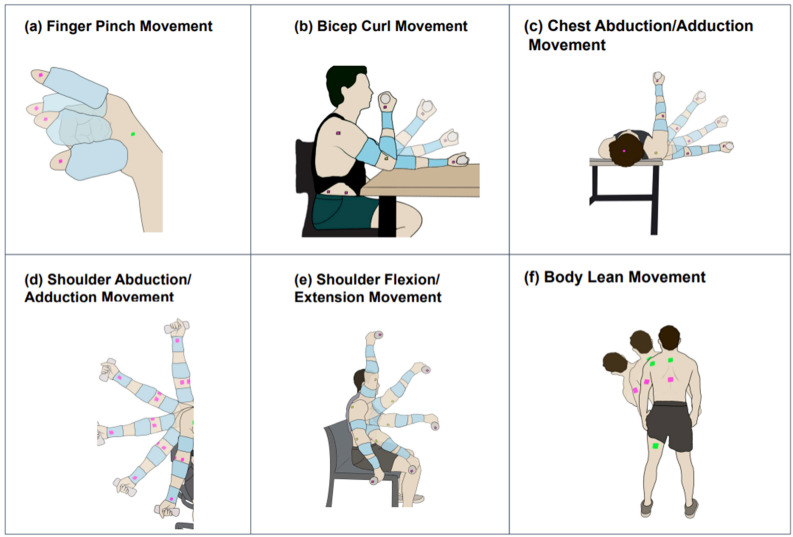
Locations of MoCa markers per six predefined motions: (**a**) finger pinch movement, (**b**) bicep curl movement, (**c**) chest abduction/adduction movement, (**d**) shoulder abduction/adduction movement, (**e**) shoulder flexion/extension movement, and (**f**) body lean movement. Sequential images outline the complete movement excursion per motion with variously colored MoCa markers indicating marker positions.

**Figure 3:**
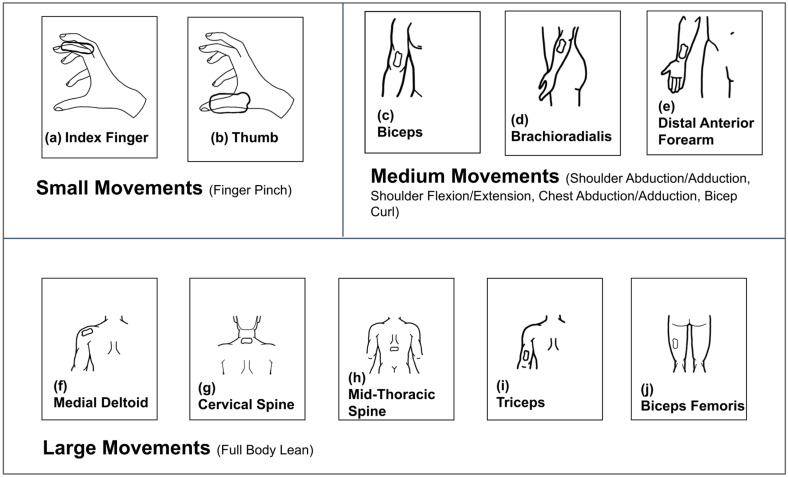
Location of BioStamp placement per motion categorized by excursion length (small, medium, and large). Small movements require two stamps: (**a**) index finger and (**b**) thumb. Medium movements require three stamps placed on (**c**) biceps, (**d**) brachioradialis, and (**e**) distal anterior forearm. Large movements require the most stamps with stamps placed on the (**f**) medial deltoid, (**g**) the cervical spine, (**h**) mid-thoracic spine, (**i**) triceps, and (**j**) biceps femoris.

To setup MoCa, a pink 2 mm marker was placed on the lateral tip of the index finger, and another pink marker was placed on the fingernail of the thumb. A green 2 mm marker was placed on the hand between the thumb and index finger (Figure 2). The finger pinch movement required two BioStamps. The BioStamps used for this movement were placed on the index finger and thumb of the subject’s dominant hand, with the tab side of the BioStamp pointing towards the tip of each finger (Figure 3). Both BioStamps were secured adjacent to the fingernail.

A table and chair were set up in the testing area, with the table directly in front of the chair. The subject was instructed to sit in a chair with a proper upright posture. The subject then rested their dominant arm on the edge of the table (Figure 2). The subject started with their fingers about 2–3 inches apart in an ‘open’ position, and when instructed to begin, performed the finger pinch for 30 s at a slow pace (1 rep/s) and a fast pace (3 reps/s) (Table 1). 

#### 2.3.2. Bicep Curl (BicepC) Movement Protocol

To setup MoCa, one 10 mm pink marker was placed on the side of the 2 lb. weight facing the camera, held in the dominant hand. The other 10 mm pink marker was placed on the deltoid muscle on the dominant side, just below the acromion. The 10 mm green marker was placed on the lateral epicondyle of the elbow on the dominant side (Figure 2). Three BioStamps were used for the bicep curl movement, placed on top of the bicep, brachioradialis, and distal anterior forearm (Figure 3).

A table and chair were set up in the testing area with the table directly in front of the chair where the subject sat. The subject sat with an upright posture, resting their dominant elbow on the edge of the table (Figure 2). The subjects started with the back of their dominant hand resting on the table. While keeping their elbow on the table, the subject performed a standard bicep curl with a 2 lb. weight in hand. They performed the bicep curl movement for 30 s at a slow pace (1 rep/6 s) and a fast pace (1 rep/2 s) (Table 1). 

#### 2.3.3. Chest Abduction/Adduction (ChestAA) Movement Protocol

MoCa required four 10 mm × 10 mm markers for the chest abduction/adduction movement: two pink, one green, and one orange. One of the pink markers was placed on the side of the dumbbell facing the camera during the movement. The other pink marker was placed on the top of the subject’s head. The green marker was placed on the top of the subject’s shoulder at the acromion on the dominant side and an orange marker was placed on the brachioradialis on the top part of the arm that was facing the camera (Figure 2). This movement required three BioStamps. They were placed on the top of the bicep, brachioradialis, and distal anterior forearm (Figure 3).

The movement (medium) was performed for 30 s at a slow pace (1 rep/6 s) and fast pace (1 rep/2 s) (Table 1). A table was set up where the subject lay on the table flat on their back. The dominant arm hung off the edge of the table straight out to the side parallel to the ground to allow a full range of movement. They extended the dominant arm out past the edge of the table. To perform this movement the subject started with their arm flat out to the side with the 2 lb. weight in their hand. When the 30 s movement period began, the subject rotated their arm by 90° to be directly in front of their body (Figure 2).

#### 2.3.4. Shoulder Abduction/Adduction (ShoulderAA) Movement Protocol

MoCa required four 10 mm × 10 mm markers for the shoulder abduction/adduction movement: two pink, one green, and one orange. One of the pink markers was placed on the medial side of the wrist. The other pink marker was placed on the front of the subject’s lateral aspect of the hip on the dominant side. The green marker was placed on the front of the subject’s deltoid muscle on the dominant side, just below the acromion, and an orange marker was placed on the elbow joint on the inside forward-facing part of the arm that was facing the camera (Figure 2). This movement required three BioStamps. The BioStamp locations were on top of the bicep, brachioradialis, and distal interior (Figure 3).

The movement (medium) was performed for 30 s at a slow pace (1 rep/6 s) and fast pace (1 rep/2 s) (Table 1). A table and chair were set up where the subject sat with proper posture. The subject started the position as described above with the arm flat down to the side of their body, pronated, with the 2 lb. weight in their hand. When the 30 s movement period began, the subject raised their arm to their side, rotating in the shoulder by 180° such that their arm was directly up in the air (Figure 2).

#### 2.3.5. Shoulder Flexion/Extension (ShoulderFE) Movement Protocol

MoCa required four 10 mm × 10 mm markers for the shoulder flexion/extension movement: two pink, one green, and one orange. The pink markers identify the endpoints of the movement, the green identifies the pivot locations, and the orange markers identify intermediate locations. One of the pink markers was placed on the side of the dumbbell facing the camera. The other pink marker was placed on the side of the lateral aspect of the hip on the dominant side. The green marker was placed on the side of the deltoid muscle on the dominant side, just below the acromion, and an orange marker was placed outside the elbow on the dominant side (Figure 2). This movement required three BioStamps. The BioStamp locations were on top of the bicep, brachioradialis, and distal anterior forearm (Figure 3).

The movement (medium) was performed for 30 s at a slow pace (1 rep/6 s) and a fast pace (1 rep/2 s) (Table 1). The subject sat in a chair with proper posture. The dominant arm hung to the side of their body with the top of the hand facing forward and the palm facing backward. When the 30 s movement period begins, the subject will raise their arm in front of themselves, rotating in the shoulder by 180° to where the arm is directly up in the air (Figure 2).

#### 2.3.6. Body Lean (BodyL) Movement Protocol

MoCa required five 10 mm × 10 mm markers for the body lean movement: two pink, two green, and one orange. All of the markers were placed directly on top of the BioStamps for this movement (Figure 2f). The pink markers were positioned on the tricep and bicep femoris BioStamps and the medial deltoid BioStamp. The green markers, identifying two pivot locations, were placed on the cervical spine and mid-thoracic spine BioStamps. This movement required five BioStamps, the most of all movements. The BioStamp locations used for this movement are on top of the medial deltoid, cervical spine, mid-thoracic spine, triceps, and bicep femoris (Figure 3).

The movement (large) was performed for 30 s at a slow pace (1 rep/8 s) and a fast pace (1 rep/4 s) (Table 1). To begin the experiment, a 10 ft × 10 ft test area was cleared and the subject stood with proper posture in the middle. When the 30 s movement period began, the subject leaned their body to the dominant side, keeping the tips of the fingers touching the side of the leg (Figure 2). The tips of the fingers moved up and down the leg as the movement was performed.

### 2.4. MoCa Statistical Methods

**Figure 4:**
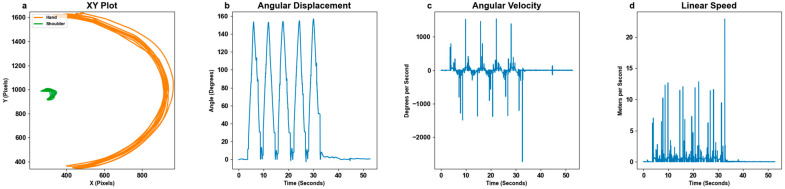
Complete data conversion process, from raw MoCa data to the final endpoint variables, subplots (**a–d**) representing data from the same trial. (**a**) Positional tracking of individual MoCa markers on the hand, elbow, and shoulder. (**b**) Angular displacement calculated by applying the cosine function to the angles formed by the markers. (**c**) Angular velocity derived from the original angles. (**d**) Linear speed calculated from positional data.

MoCa data were collected via video recordings (.mp4 or .mov) which were gathered and renamed for convenience. The MoCa markers were tracked using a color detection program on each frame of the video which outputted coordinate data in pixels. Angles (degrees) were found between two or three markers. To obtain angles from three markers, the standard law of cosine was applied. To obtain angles from two markers, the initial position of the non-pivot marker was used as a “ghost point” to mimic a third stamp position. Next, the law of cosines was used to find the angle between the two tracked markers and the new “ghost point”. Because the law of cosines only calculates the absolute angle, the resulting angle was manually inverted when the moving marker crossed its original position in x/y, as it represents a negative displacement from the initial angle. The derivative of the angular displacement outputted angular velocity (degrees per second). Linear speed was calculated by determining the displacement of the markers over time. First, each pixel recorded was converted into meters using manual measurements for each subject’s trial. Multiple measurements were manually recorded via a tape measure. The distance in meters between each stamp was recorded for each trial. These measurements were then used to scale the pixels to meters. Displacement was computed as the Euclidian distance between consecutive positions in meters. This displacement was then divided by the time difference between frames to yield linear speed in meters per second (m/s). The full MoCa statistical steps are shown in Figure 4. In addition to the full motion data, the maximum angular displacement was calculated by averaging the top 5% of the highest angular displacements values.

### 2.5. BioStamp Statistical Methods

**Figure 5:**
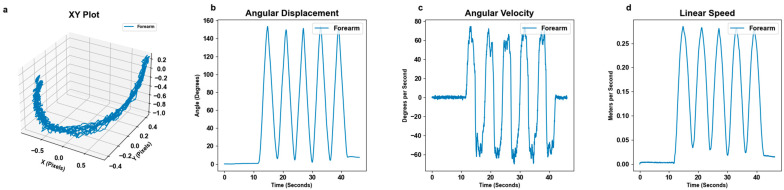
Full data conversion steps from obtaining raw BioStamp data into the final endpoint variables subplots (**a**–**d**) are from the same trial. (**a**) Marker tracking collected along the X, Y, and Z axes in pixels. (**b**) Angular displacement obtained through further integration. (**c**) Angular velocity integrated from BioStamp’s output acceleration data. (**d**) Linear speed obtained from acceleration output using 3-dimensional Pythagorean theorem.

Raw data collected from BioStamps were uploaded to the MC10 BioStamp website where files are stored. We downloaded and renamed them for convenience. Each BioStamp outputs three axes of angular velocity (°/s) and linear acceleration (g) [25]. We gathered angular velocity from the BioStamp marker that moved the most, as it captured the most relevant movement in reference to the motion at hand. To enhance the quality and accuracy of the gyroscope angular velocity measurements, a low-pass Butterworth filter was applied. The purpose of this filtering step is to make angular velocity comparable to MoCa by reducing high-frequency noise that can obscure the true signal of interest. Acceleration data inherently capture the constant force of gravity. In order to isolate gravitational acceleration from raw acceleration data, we applied a low-pass filter with a smoothing factor α=0.9. The gravitation component at each time step was calculated by incorporating the previous gravity estimate with the current acceleration data. This resulting gravity vector accounts for changes in sensor orientation and is subtracted from the raw acceleration to obtain linear acceleration [26]. The 3-dimensional linear speed of the sensor (m/s) was calculated by integrating the acceleration in each axis ($m/s^{2}$) over time and then computing the Euclidian norm of the resulting vectors across each time interval [27]. For the X, Y, and Z axes on each BioStamp, angular velocity (°/s) was integrated to obtain angular displacement (°). The full data conversion process and each metric at each step are shown in Figure 5. In addition to the full motion data, the maximum angular displacement was calculated by averaging the top 5% of the highest angular displacements values.

### 2.6. Comparison Methods

After completing the full steps of the experiment and obtaining the raw data, with the steps outlined in Figure 1, comparisons and correlations between the two systems were determined.

#### 2.6.1. Maximum Angular Displacement Comparison

Maximum angular displacement was calculated to determine the range of motion. For both MoCa and BioStamp, maximum angular displacement values were calculated by averaging the top 5% of the highest angular displacement values. Direct comparisons between averages were made.

#### 2.6.2. Alignment of Trials

To determine agreement between MoCa and BioStamp, each corresponding trial between BioStamp and MoCa must first be aligned to provide corresponding measurements. Resampling using linear interpolation was used to match the sample rate between systems for accurate comparisons [28]. The datasets were offset and trimmed to the same start time; however, MoCa can only give epoch time information to the nearest second of accuracy, whereas the BioStamp has millisecond accuracy, leading to possible misalignment between the two. To account for that, cross correlations were collected to find the highest correlation value offset for each one.

#### 2.6.3. Bland–Altman Analysis

To compare MoCa and BioStamp, Bland–Altman analysis (a form of Tukey mean-difference plot) was utilized to determine agreement as well as systemic and relative bias. We first conducted the Bland–Altman analysis on a motion-by-motion scale to assess relative bias on angular displacement, angular velocity, and linear speed. Secondly, we performed the analysis on the three endpoint metrics across all trials to assess systemic bias. In either case, the premise outlined here for analysis was utilized.

Bland–Altman analysis is typically represented as a scatter plot where the difference between system A and system B are plotted against the mean measurement plotted [29]. The mean difference can then be interpreted as the average bias of system A relative to system B. Limits of agreement are calculated to visually display the typical variation in the differences, with wider limits of agreement indicative of disagreement between systems, and narrower limits of agreement indicative of better alignment. Given a normal distribution of differences, 95% of differences are expected to be within the limits of agreement given by $\bar{d}\pm 1.96\times \mathrm{SD}$, where $\bar{d}$ is the mean difference and the SD is the standard deviation of the differences [29].

Without a normal distribution of the differences, the limits of agreement become less reliable. In any of the analyses conducted in this experiment, the sample size for any Bland–Altman plot becomes well over 100,000 data points, given the multitude of measurements for any trial. As such, traditional methods for assessing normal distribution become less reliable. Therefore, the distribution of differences was visually inspected for normality.

If the relationship between the differences and the magnitude of the data is complicated, then the difference may be modeled as proportional to the magnitude of the measurement, given such a trend exists [30]. A linear regression line of best fit was modeled for any analysis. The extremely large sample size in any case (>100,000) makes the *p*-value of a linear regression unreliable. Therefore, we predetermined an r-squared value of above 0.5 to signify a significant enough relationship. We then regress the absolute residuals from the first linear regression to the magnitude of the data. For the reasons outlined above, a significant relationship is again determined by an r-squared value above 0.5. In which case, the limits of agreement are determined to incorporate both regressions, D^ and R^, as per Formula (1), following established guidelines.

(1) Limits of Agreement=D^±1.96π2R^

In the case that the relationship between the absolute residuals and the differences is not significant, then the limits of agreement are determined with the standard deviation, SD, of the residuals of the line of best fit, D^, as shown in Formula (2), following established guidelines.

(2) limits of Agreement=D^±1.96SD

In any case, each trial was aligned as per the steps outlined in Section 2.6.2. Then, the distribution of differences between MoCa and BioStamp were assessed. Given that a distribution of differences was not normal and there was not a significant linear relationship, as determined by the r-squared value, we used nonparametric methods for assessing agreement. Though nonparametric evaluations are not as reliable as parametric evaluations given a small sample size, we circumvent such a limitation given our large sample size in any case. The upper and lower bounds of agreement were given within the 5% percentile and 95% percentile of data, providing 90% of the differences within their bounds.

#### 2.6.4. Relative Limits of Agreements

In order to accurately compare the mean difference and limits of agreement between MoCa and BioStamp, the results of the Bland–Altman analysis had to be normalized to a given movement. Angular displacement limits of agreement would be normalized to the angles typically found for a given movement. Specifically, the upper limit of agreement would be normalized to the typical maximum angle, as both provide a non-negative descriptor for a motion. Moreover, the upper limit of agreement is an equal distance away from the average difference compared to the lower limit of agreement, and as such is the expected case for the maximum angular displacement relative to its minimum angular displacement.

The agreement efficiency for a motion is calculated as the upper limit of agreement relative to the maximum angular displacement, as per Formula (3). By taking the inverse of the ratio of the upper limit of agreement to its maximum angle, a higher value indicates improved agreement efficiency. This transformed allows for a more straightforward understanding of the alignment between MoCa and BioStamp.

(3) $$\mathrm{Agreement}\;\mathrm{Efficiency}\;(\%)=(1-\frac{\mathrm{Upper}\;\mathrm{Limit}\;\mathrm{of}\;\mathrm{Agreement}}{\mathrm{Maximum}\;\mathrm{Angle}})\times 100$$

#### 2.6.5. 1-to-1 Correlations

Correlations between MoCa and BioStamp were drawn for several measurements. Angular displacement correlations and angular velocity correlations between the two systems were gathered using similar processes. Resampling and alignment were first conducted as outlined in Section 2.6.2. Pearson correlation coefficients were then calculated for corresponding datasets.

Linear speed correlations required comparing the 2-dimensional MoCa speed and 3-dimensional BioStamp speed. For MoCa, the distance one marker moved in two dimensions, between two frames divided by the time elapsed, produced linear speed. For BioStamp, the linear speed was calculated by dividing the distance traveled by the BioStamp in three dimensions between two sample times by the elapsed time. The two datasets were resampled, trimmed, and offset using the same process as described above. At that point, the linear speed between systems could be accurately correlated.

#### 2.6.6. Aggregate Bar Charts

For each 1-to-1 correlation, a one-line summary was written to a data aggregate file containing the information particular to that file such as the motion, subject, camera angle, trial number, and speed of trial as well as the angular displacement correlation, the angular velocity correlation, and the linear speed correlation. The bar charts were created by grouping the aggregate data by motion and/or speed and then finding the mean of each group. The bar charts created were the average angular displacement correlation per motion, the average angular displacement correlation per motion and speed, the average angular velocity correlation per motion, the average angular velocity correlation per motion and speed, the average linear speed correlation per motion, and the average linear speed correlation per motion and speed.

## 3. Results

### 3.1. Motion Tracking

**Figure 6:**
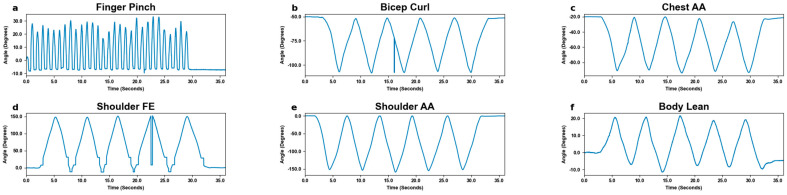
MoCa−captured angular displacement for all six motions: (**a**) finger pinch, (**b**) bicep curl, (**c**) chest abduction/adduction, (**d**) shoulder flexion/extension, (**e**) shoulder abduction/adduction, and (**f**) body lean.

**Figure 7:**
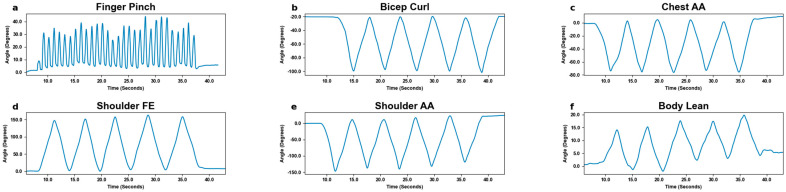
BioStamp−captured angular displacement for all six motions: (**a**) finger pinch, (**b**) bicep curl, (**c**) chest abduction/adduction, (**d**) shoulder flexion/extension, (**e**) shoulder abduction/adduction, and (**f**) body lean.

MoCa and BioStamp each demonstrated clear ability and utility to track movements and motion. MoCa tracked movement on a marker basis via position, while BioStamp tracked movement on a stamp basis via gyroscopic tracking. Both MoCa and BioStamp effectively tracked angular displacement in reference to the body angle of the motion displayed. MoCa angular displacement graphs across all motions (Figure 6) showed the pattern of motion reps, demonstrating consistent tracking. The BioStamp system was able to track the defined identical movements (Figure 7) with similar angular displacement patterns.

### 3.2. Motion Detection by Excursion Size

**Figure 8:**
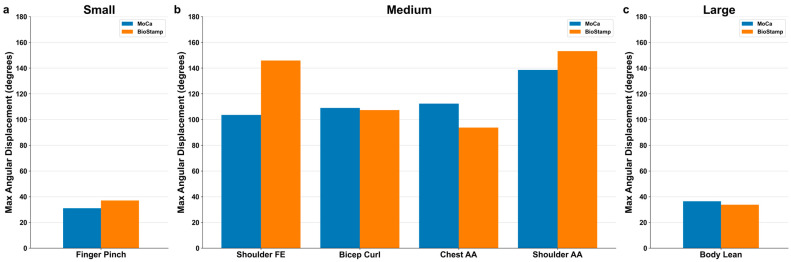
Average maximum angular displacement per motion categorized by motion size: (**a**) small, (**b**) medium, and (**c**) large. MoCa (blue) and BioStamp (orange) are compared side by side.

The six predefined motions were categorized by excursion size (i.e., small, medium, and large). The average maximum angular displacement across all motions is presented in Figure 8. The small motion category consisting of finger pinch resulted in an average maximum angular displacement of 31.05° for MoCa and 37.08° for BioStamp. The medium motion category consisted of shoulder flexion/extension, bicep curl, chest abduction/adduction, and shoulder abduction/adduction. The average maximum angular displacement for shoulder flexion/extension was 103.60° for MoCa and 145.93° for BioStamp. Bicep curl was 108.97° for MoCa and 107.38° for BioStamp. Chest abduction/adduction was 112.35° for MoCa and 93.71° for BioStamp. Shoulder abduction/adduction was 138.57° for MoCa and 153.20° for BioStamp. The large motion category consisted of body lean. The average maximum angular displacement for body lean was 36.46° for MoCa and 33.78° for BioStamp.

**Figure 9:**
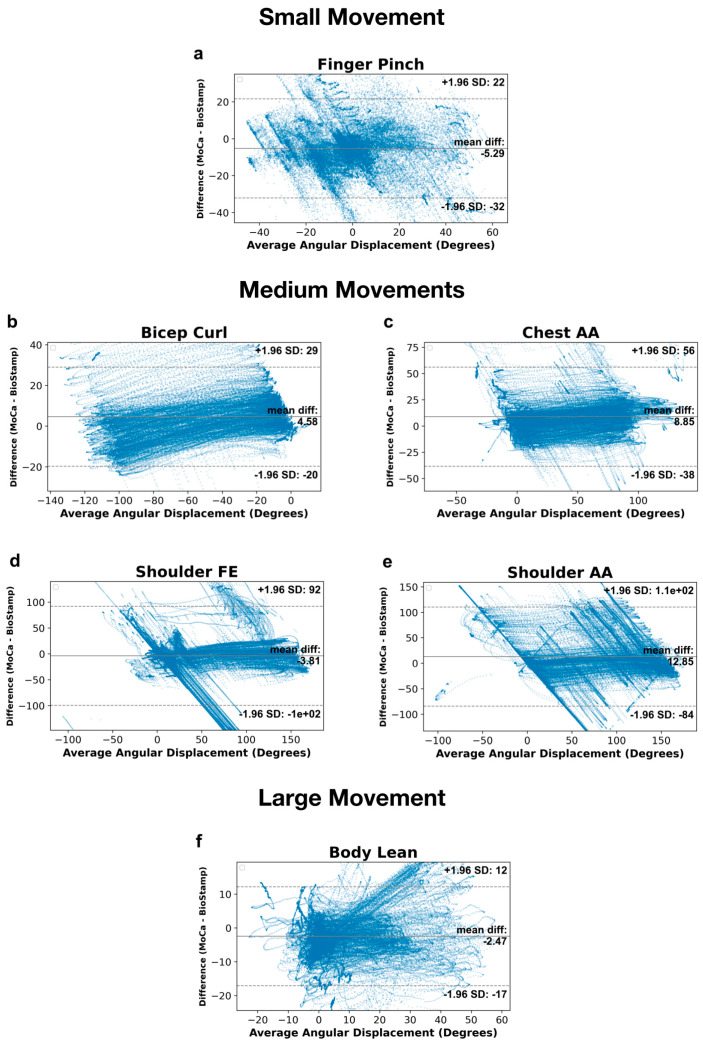
Bland−Altman plots of angular displacement between MoCa and BioStamp per motion categorized by motion size: small, medium, and large. (**a**) Bland−Altman plot of finger pinch movement. (**b**) Bland−Altman plot of bicep curl movement. (**c**) Bland−Altman plot of chest abduction/adduction movement. (**d**) Bland−Altman plot of shoulder flexion/extension movement. (**e**) Bland−Altman plot of shoulder abduction/adduction movement. (**f**) Bland−Altman plot of body lean movement.

The Bland–Altman analysis comparing MoCa and BioStamp angular displacement values is presented in Figure 9, organized by motion type. Figure 9a displays the Bland–Altman analysis of the finger pinch motion, the mean difference between MoCa and BioStamp is −5.29 degrees, with 95% limits of agreement ranging from −32.13 to 21.56 degrees. Figure 9b presents the analysis of the bicep curl motion, with a mean difference of 4.58 degrees and the 95% limits of agreement ranging from −19.78 to 28.94 degrees. The chest abduction/adduction motion is represented in Figure 9c, with a mean difference of 8.85 degrees and the 95% limits of agreement ranging from −38.4 to 56.1 degrees. The shoulder flexion/extension motion is analyzed in Figure 9d, with a mean difference of −3.81 degrees and the 95% limits of agreement ranging from −99.6 to 91.98 degrees. Figure 9e focuses on the analysis of the shoulder abduction/adduction motion, with a mean difference of 12.85 degrees and the 95% limits of agreement ranging from −84.46 to 110.16 degrees. Finally, the body lean motion is displayed in Figure 9f, with a mean difference of −2.47 degrees and the 95% limits of agreement ranging from −17.11 to 12.17 degrees.

**Figure 10:**
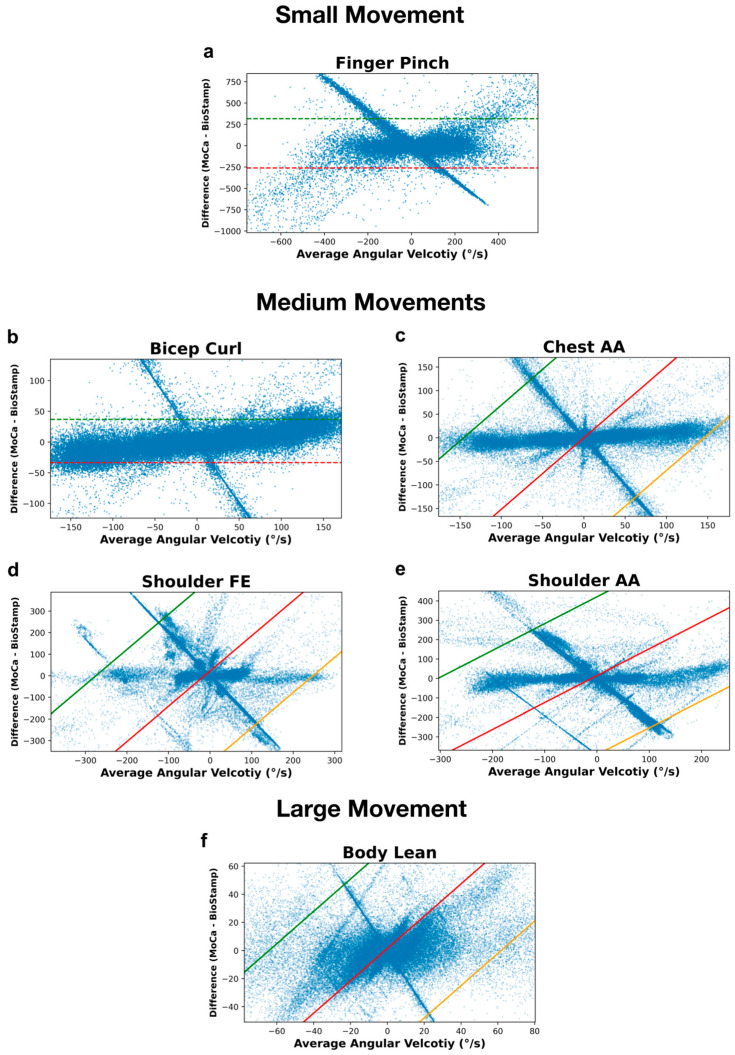
Bland−Altman plots of angular velocity between MoCa and BioStamp per motion categorized by motion size: small, medium, and large. (**a**) Bland−Altman plot of finger pinch movement with the 5% percentile of differences denoted by the red line, and the 95% percentile of differences denoted by green line. (**b**) Bland−Altman plot of bicep curl movement with the 5% percentile of differences denoted by the red line, and the 95% percentile of differences denoted by green line. (**c**) Bland−Altman plot of chest abduction/adduction movement, with the linear regression shown in red, the lower limit of agreement in yellow, and the upper limit of agreement in green. (**d**) Bland−Altman plot of shoulder flexion/extension movement, with the linear regression shown in red, the lower limit of agreement in yellow, and the upper limit of agreement in green. (**e**) Bland–Altman plot of shoulder abduction/adduction movement. (**f**) Bland−Altman plot of body lean movement, with the linear regression shown in red, the lower limit of agreement in yellow, and the upper limit of agreement in green.

The motion-by-motion analysis of angular velocity values between MoCa and BioStamp are presented in Figure 10. Figure 10a displays the Bland–Altman analysis of the finger pinch motion, the range between the fifth percentile and ninety-fifth percentile of differences being between −259.98 degrees per second and 316°/s, respectively. Figure 10b presents the analysis of the bicep curl motion, with the bottom fifth percentile of differences at −33.48°/s and the ninety-fifth percentile of differences at 36.86°/s. The chest abduction/adduction motion is represented in Figure 10c, with the difference represented by a linear relationship having a y-intercept of—0.05°/s, and a slope of 1.52. The 95% limits of agreement are relative to this regression, with y-intercepts at −221.03 and 220.93°/s. The shoulder flexion/extension motion is analyzed in Figure 10d, with the difference represented by a linear relationship having a y-intercept of 21.99°/s and a slope of 1.63. The 95% limits of agreement follow this trend, with y-intercepts at −404.22 and 448.2°/s. Figure 10e focuses on the analysis of the shoulder abduction/adduction motion, with the difference represented by a linear relationship having a y-intercept of 14.34°/s and a slope of 1.38. The 95% limits of agreement align with this trend, with y-intercepts at −392.4 and 421.07°/s. Finally, the body lean motion is displayed in Figure 10f, with the difference represented by a linear relationship having a y-intercept of 1.41°/s and a slope of 1.15. The 95% limits of agreement follow this trend, with y-intercepts at −71.04 and 73.87°/s.

**Figure 11:**
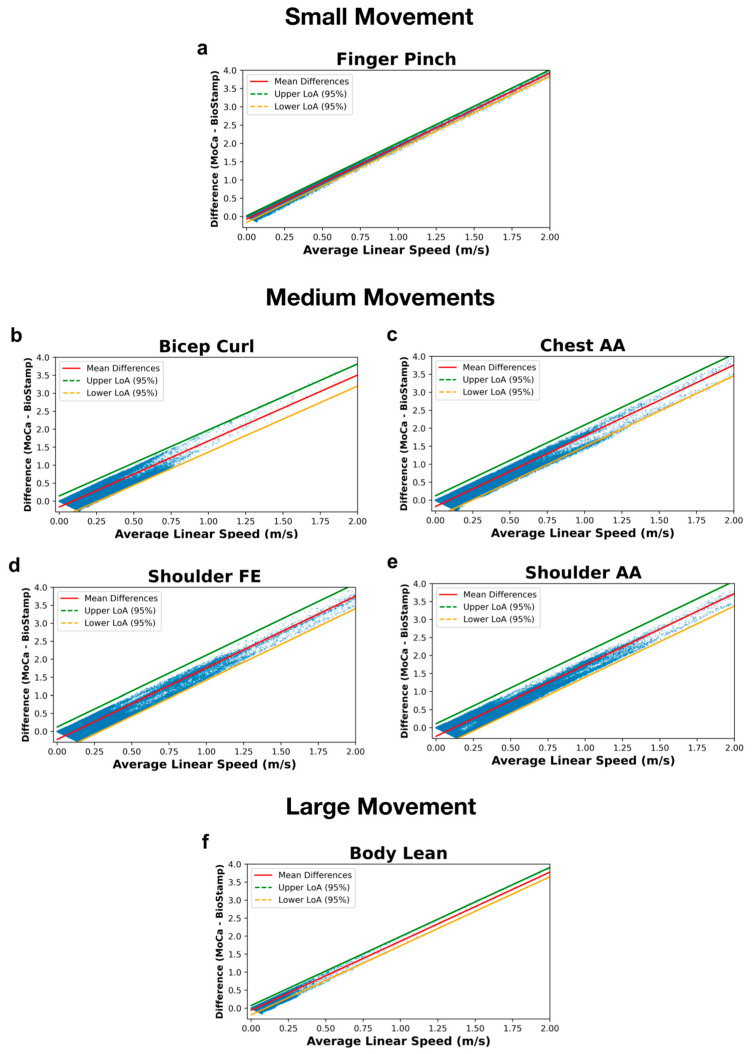
Bland−Altman plots of between MoCa and BioStamp per motion categorized by motion size: small, medium, and large. Blue points indicate single measurement pairs between MoCa and BioStamp. The red line indicates the linear regression, with the lower limit of agreement in yellow, and the upper limit of agreement in green. (**a**) Bland–Altman plot of the finger pinch movement. (**b**) Bland–Altman plot of the bicep curl movement. (**c**) Bland–Altman plot of the chest abduction/adduction movement. (**d**) Bland–Altman plot of the shoulder flexion/extension movement. (**e**) Bland–Altman plot of the shoulder abduction/adduction movement (**f**) Bland–Altman plot of the body lean movement.

Figure 11 displays the linear speed analysis between MoCa and BioStamp via Bland–Altman plots grouped by motion type. Figure 11a displays the Bland–Altman analysis of the finger pinch motion, with the difference represented by a linear relationship having a y-intercept of −0.07 m/s and a slope of two. The 95% limits of agreement follow the same slope as the linear regression, with y-intercepts at −0.16 and 0.02 m/s. Figure 11b presents the analysis of the bicep curl motion, with the difference represented by a linear relationship having a y-intercept of −0.16 m/s and a slope of 1.84. The 95% limits of agreement are in respect to the linear relationship, with y-intercepts at −0.47 and 0.14 m/s. The chest abduction/adduction motion is represented in Figure 11c, with the difference represented by a linear relationship having a y-intercept of −0.18 m/s and a slope of 1.97. The 95% limits of agreement followthis linear trend, with y-intercepts at −0.48 and 0.12 m/s. The shoulder flexion/extension motion is analyzed in Figure 11d, with the difference represented by a linear relationship having a y-intercept of −0.22 m/s and a slope of 1.99. The 95% limits of agreement follow this linear trend, with y-intercepts at −0.57 and 0.12 m/s. Figure 11e focuses on the analysis of the shoulder abduction/adduction motion, with the difference represented by a linear relationship having a y-intercept of −0.24 m/s and a slope of 1.98. The 95% limits of agreement follow the slope of the linear trend, with y-intercepts at −0.59 and 0.11 m/s. Finally, the body lean motion is displayed in Figure 11f, with the difference represented by a linear relationship having a y-intercept of −0.06 m/s and a slope of 1.92. The 95% limits of agreement follow this linear trend, with y-intercepts at −0.19 and 0.07 m/s.

**Figure 12:**
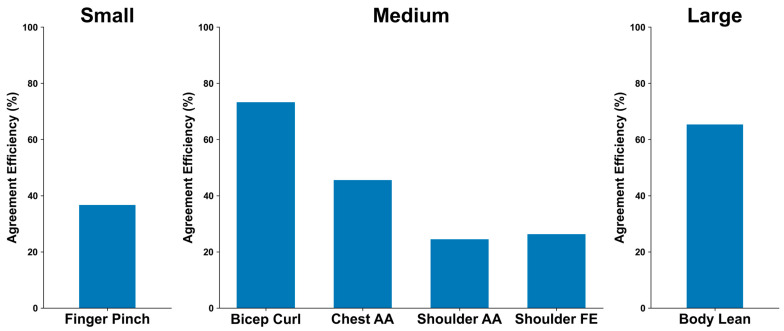
Agreement efficiency (%) for each motion, grouped by motion size categories, small, medium, and large.

Figure 12 illustrates the relative limits of agreements for angular displacement between MoCa and BioStamp across the movement sizes. The finger pinch motion is the only motion in the small movement category with an agreement efficiency of 36.71%. In the middle movement category are bicep curl, with an agreement efficiency of 73.24%, chest abduction/adduction, with an agreement efficiency of 45.55%, shoulder abduction/adduction at 24.49%, and shoulder flexion/extension at 26.28%. Body lean is the sole motion in the large movement category, with an agreement efficiency of 65.35%.

### 3.3. Motion Detection Endpoint Variables

**Figure 13:**
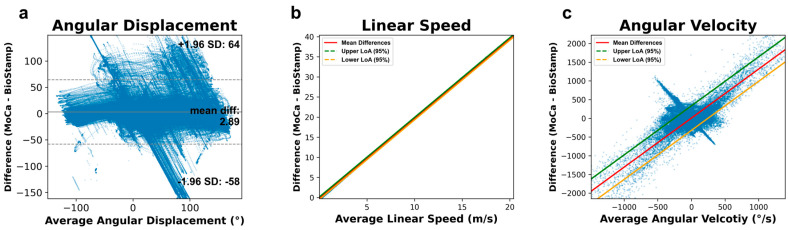
Bland−Altman plot between MoCa and BioStamp across endpoint variables. (**a**) Bland–Altman plot of angular displacement values. (**b**) Bland−Altman plot of linear speed values. (**c**) Bland–Altman plot of angular velocity values.

Figure 13 illustrates the Bland–Altman analysis between MoCa and BioStamp across all trials. Figure 13a illustrates the comparison of angular displacement, with a mean difference of −2.89 degrees, and the 95% limits of agreement of −58.3 and 64.08 degrees. Figure 13b demonstrates linear speed between MoCa and BioStamp, with a linear relationship between the meters per second difference and the average meter per second recorded. The slope of the relationship is 1.98 with a y-intercept of −0.16 m/s. The 95% limits of agreement are in respect to this linear trend, with y-intercepts at −0.47 and 0.15 m/s. The comparison of angular velocity is represented in Figure 13c, with a linear regression illustrating the degree per second difference relative to the average angular velocity. The y-intercept of this relationship is 5.3 degrees per second with a slope of 1.31. The 95% limits of agreement are in respect to this linear relationship, with y-intercepts at −332.44 and 333.04 degrees per second.

**Figure 14:**
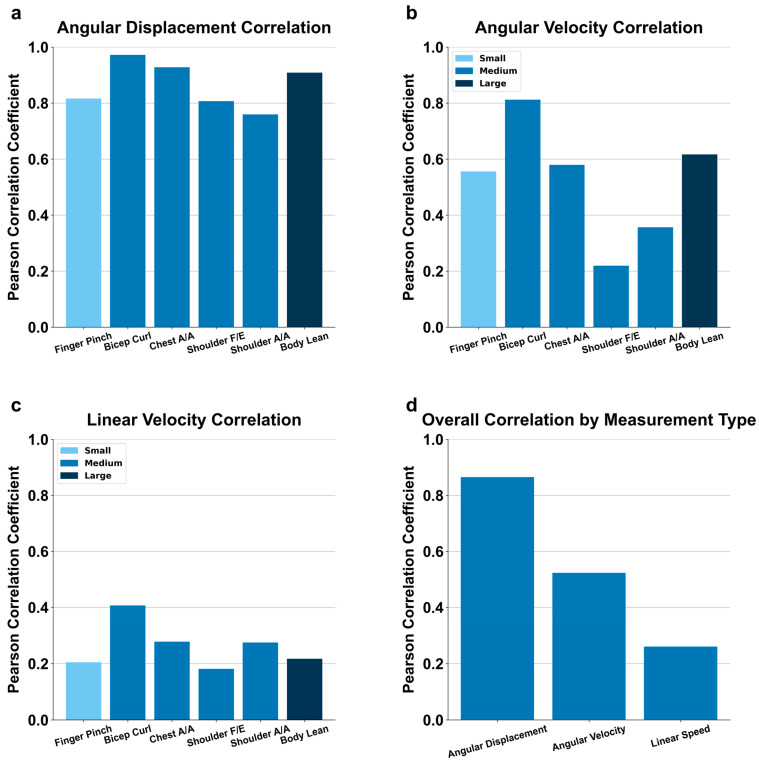
(**a**) Pearson correlation coefficients for angular displacement between MoCa and BioStamp across all predefined motions. (**b**) Pearson correlation coefficients for angular velocity between MoCa and BioStamp across all motions. (**c**) Pearson correlation coefficients for linear speed between MoCa and BioStamp across all motions. (**d**) Average Pearson correlation coefficients for MoCa versus BioStamp across three metrics: angular displacement, angular velocity, and linear speed.

Figure 14a illustrates the angular displacement correlations between MoCa and BioStamp across all predefined motions. The bicep curl exhibited the highest angular displacement correlation between the two systems at 0.972, followed by chest abduction/adduction at 0.928. Body lean, representing a large movement, had the third-highest correlation at 0.909. Finger pinch, a small movement, showed a correlation of 0.816. Shoulder flexion/extension and shoulder abduction/adduction had correlations of 0.807 and 0.760, respectively. Overall, all motions produced relatively high correlation values when comparing MoCa and BioStamp.

Angular velocity correlations between MoCa and BioStamp are presented in Figure 14b. The highest angular velocity correlation was observed for the bicep curl at 0.812. Body lean and chest abduction/adduction followed with correlations of 0.617 and 0.579, respectively. Finger pinch showed a correlation of 0.556, while shoulder abduction/adduction and shoulder flexion/extension exhibited lower correlations of 0.356 and 0.219, respectively.

Linear speed correlations between MoCa and BioStamp are displayed in Figure 14c. The bicep curl demonstrated the highest average linear speed correlation at 0.406. Other motions showed lower correlations: body lean (0.217), chest abduction/adduction (0.278), finger pinch (0.204), shoulder abduction/adduction (0.275), and shoulder flexion/extension (0.181). The overall average linear speed correlation across all motions was 0.260.

Figure 14d presents the overall correlation metrics between MoCa and BioStamp across three primary measurements. Angular displacement showed the highest correlation at 0.865, followed by angular velocity at 0.523, and linear speed at 0.260.

### 3.4. Motion Detection by Speed

**Figure 15:**
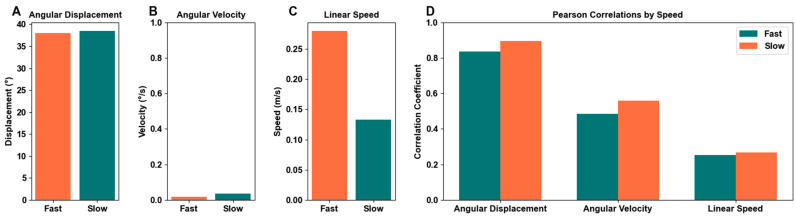
Comparison between slow (orange) and fast (teal) runs. (**A**) Average angular displacement comparison. (**B**) Average angular velocity comparison. (**C**) Average linear speed comparison. (**D**) Pearson correlation coefficients for angular displacement, angular velocity, and linear speed.

Figure 15 shows the average angular displacement (a), angular velocity (b), linear speed (c), and average Pearson correlation coefficients delineated by speed (d). The average angular displacement, which includes all motions and recording modes, shows the fast motions with an average 38.038 degrees and the slow motions at 38.523, a 0.485 degree difference between the speeds. The average angular velocity shows the fast motions at 0.017 degrees per second and the slow motions at 0.037 degrees per second, a 0.020 dps difference. The average linear speed records the fast motions at 0.280 m/s and the slow motions at 0.133 m/s, a 0.147 m/s difference. The correlations between three metrics show a slight disparity between speeds, with slower speeds correlating consistently as equal to or higher than the fast speeds. The average angular displacement correlation for fast speeds is 0.835, and for slow speeds is 0.895. The average angular velocity correlation for fast speeds is 0.486, and for slow speeds is 0.560. The average linear speed correlation for fast speeds is 0.253, and for slow speeds is 0.268. On average, slow speeds recorded 9.47% higher correlations than the fast speeds.

## 4. Discussion

Motion analysis is a critical tool for monitoring body movement as an element of human health, providing important insights as to functional status versus the presence, evolution, or recovery from movement-affecting disease [13,23,31]. The impairment of everyday motions may be an early indicator of changing health [13,25,32]. Motion analysis quantifies movement and is useful to non-invasively identify abnormalities associated with a wide range of diseases [13,17,33,34]. Our study shows that both the MoCa system and the BioStamp allow for a quantitative assessment of a range of human movements of varying excursions. Further, our study shows that each system has its own identifiable differences corresponding to unique advantages and disadvantages.

### 4.1. Trackability

Six predefined motions were tracked using MoCa and BioStamp to analyze comparable output data for the same identical movements. Maximum angular displacement was calculated for each dataset to analyze the maximum extent of motion exerted in each trial. Figure 8 demonstrates very comparable degrees of maximum angular displacement between systems. Half of the tested motions (bicep curl, chest abduction/adduction, and body lean) had higher averages in MoCa, while the other motions (finger pinch, shoulder flexion/extension, and shoulder abduction/adduction) had higher averages in BioStamp.

Though both systems were being used to track the same motion, the placement of MoCa markers and BioStamps were in slightly different physical locations on the body for each motion, leading to slight differences between MoCa and BioStamp. MoCa markers can be placed anywhere and thus were placed on strategic pivot points and limbs to effectively and optimally capture the fullest motion and discernible angles. BioStamps have placement guidelines per motion (MC10 cloud). The slight placement discrepancy between the BioStamp and MoCa markers may have caused higher maximum angular values in MoCa than in BioStamp.

In our trials, we observed systematic trends where the BioStamp displacement exhibited gradual shifts either upward or downward over time, while the overall range of motion remained constant. BioStamp’s angular displacement was derived by integrating the angular velocity measured by the gyroscope. These shifts suggest potential gyroscopic sensor drift, a known issue where error accumulation occurs in the measurement of angular velocity and is thus reflected in the angular displacement trends [35]. Although the system successfully tracked the six predefined motions, additional post-processing corrections are necessary to enhance the accuracy of the displacement data [36].

There was very obvious noise in MoCa’s linear speed, as seen in the linear speed Bland–Altman analysis. The proportional bias in any case suggests a systemic issue wherein MoCa generally overestimates at larger linear speeds at any point. MoCa produced angular displacement results that were well aligned to BioStamp, which is supportive evidence that the system does not have trouble tracking motion, but rather the linear speed noise may be indicative of a large sensitivity to movements of the markers.

Overall, the detection of the tested motions was successful using each system. Varying values of angular displacement detected can be attributed to MoCa marker placement or sensor drift inherent to BioStamp. Despite the minor variance, both MoCa and BioStamp effectively captured the motion’s extent, shape, and range with good overall correlation, though some movements correlated to a greater degree than others.

### 4.2. Motion Size Comparison

Size categories were created across the six motions to examine the ability of each system to quantitate motion across differing motion excursion ranges, i.e., sizes, defined in terms of dimensional areas, i.e., as small (<1 sq ft), medium (1–5 sq ft), and large (6–10 sq ft).

#### 4.2.1. Small Motion

Finger pinch was the sole motion in the small motion size category. The small scale of the finger pinch motion did not affect tracking ability and both MoCa and BioStamp were able to track the motion.

The negative mean difference in the angular displacement indicates that MoCa generally produced angles lower than BioStamp. Though the Pearson correlation coefficient was large, the low agreement efficiency indicates that there was a very significant discrepancy between the two systems in terms of angular displacement. Angular velocity between the two systems varied greatly, though it did not exhibit a significant linear relationship between differences and velocity as was the case for other motions. Linear speed had a very strong correlation, with a slope of two. As seen in many examples, it was clear that finger pinch exhibited a large degree of noise in terms of speed. The small motion size may have contributed to the error found within the comparisons. As a suggested use case, twitching and sudden small muscular spasms can arise as signs of chronic kidney disease (CKD) [37]. The finger pinch experiment illustrates that MoCa and BioStamp should go through further accuracy training before being used to assess sudden and small muscular spasms.

#### 4.2.2. Medium Motions

Correlations varied over a spectrum within the medium size motion category, comprising bicep curl, chest abduction/adduction, shoulder abduction/adduction, and shoulder flexion/extension.

Bicep curl had the most aligned angular displacements between systems, indicated by their very high correlation coefficients and agreement efficiencies. The positive mean difference in the angular Bland–Altman analysis indicates that MoCa tended to produce larger angles than BioStamp. It also had the narrowest angular velocity limits of agreement, indicating a greater agreement of velocity than in other motions. The stark slope in the linear Bland–Altman analysis indicates very strong proportional bias.

The chest abduction/adduction movement was aligned between systems better than some motions and worse than others. The angular results indicated that the differences between systems were half that of the observed values, with MoCa having relatively higher angles on average. The stark regressions in both linear speed and angular velocity represent a proportional bias for speed measurements.

Shoulder flexion/extension as well as shoulder abduction/adduction had the lowest agreement between systems compared to all other motions. Both had limits of agreement that were roughly 75% of the total angles for their motions. MoCa tended to produce lower angles than BioStamp for shoulder FE while it tended to produce much larger values for shoulder AA. Both motions had very evident proportional bias in terms of speed, as indicated by the significant linear trends.

As an example of potential use, tracking limb movement in restless leg syndrome (RLS) which is common in chronic kidney disease patients, would be useful in diagnosis and treatment [38]. The medium motion studies demonstrate the ability of both MoCa and BioStamp to track erratic movements in a limb, as occurs in RLS, though addressing systemic noise could benefit the use of either system in such cases.

#### 4.2.3. Large Motion

Body lean was the sole motion in the large size motion category and had alignment between systems second best only to the bicep curl motion. The angular displacement comparisons indicate a large correlation and agreement, with a very slight bias for MoCa to produce lower angles. The speed analysis indicate a proportional bias.

The large size category represents motion tracking for near full-body motions. CKD can cause involuntary movements as well as bodily fatigue that can be apparent on a greater area of the patient’s body [39,40]. As such, MoCa’s and BioStamp’s ability to track body lean’s larger size demonstrates the accuracy in large-scale motion abnormality detection for both systems.

#### 4.2.4. Size Definitions

The size definition had seemingly no correlation with how well the motions tracked. Across agreement efficiency, Pearson correlations, and maximum angular displacement, the most observable differences between systems are generally found for the shoulder movements, both belonging to the medium category. Though the bicep curl and body lean motion belonged to different motion size categories, they generally tracked the best and aligned the most between systems. The mean difference bias across motions had no correlation to the size category either, given that three of the medium motions had larger angles for MoCa than BioStamp, with the other three motions having lower angles for MoCa. The speed results indicated a proportional bias for every motion save for the finger pinch and bicep curl angular velocity. Bicep curl’s angular velocity Bland–Altman analysis was the only speed analysis that had even remotely comparable differences between MoCa and BioStamp, which makes sense when considering that the angular results were best for the motion.

The agreement efficiency normalizes the differences between systems relative to the typical angles for the motion. That makes the statistic a comparable one, in which case there is a slight trend, with the small motion having the worst agreement, the medium movements having generally better agreement, and the large movement having better agreement. The limitation of analyzing such a trend is the lack of multiple motions in the small and large motion category. Nonetheless, we can estimate that the larger a motion is, as defined by the terms in the paper, the more room for error and thus, the accuracy of the motion. For example, the intricate movements of a finger pinch require the minimal movement of the fingers, whereas the body lean motion requires a full-body tilt. Such disparities in the motions could lend themselves to tracking ability.

It may be worth noting that the difference in agreement between motions in the medium motion category may suggest that motion size alone is not responsible for tracking ability. The motions that had greater MoCa tracking failure were on muscles that have a greater axis of rotation, such as the shoulders. Revisiting Figure 3e shows the markers placed on parts of the arm with a greater chance of twisting away from camera view, such as the shoulder and elbow. The motions may have been more difficult to perform because the subjects did not have an armrest for their arms, which lent itself to more arm swiveling. Additionally, some variance in the orientation of the limb could be explained by the joints’ greater range of movement while performing the motion. The motions that performed the best had the best lateral view of all markers. All of the motions tested had the proven ability to have all MoCa markers in view of the camera as MoCa can only track in two dimensions. However, the tendency of the limbs to twist markers out of frame is greater for some motions than others, accounting for some performance reduction.

### 4.3. Endpoint Variables

We compared the efficacy of each system across motion endpoint variables. The Bland–Altman analysis would thereby display any systemic or relative bias.

The Bland–Altman analysis of angular displacement across all trials displayed a mean difference of 2.89, which indicates a very small systemic bias, where MoCa generally produces angles on average 2.89 degrees larger than BioStamp. This indicates that MoCa tends eto produce angles similar to BioStamp, though the wide limits of agreement indicates variability in differences. This indicates relative bias as opposed to a systemic bias on the behalf of MoCa, given that the mean difference differs from motion to motion. The Pearson correlation coefficient of 86.5% indicates a strong alignment of trends between the systems. Variation in correlation coefficients may be attributed to MoCa’s loss of a marker during tracking. When a MoCa marker’s position in the video is lost, its coordinates are static until its position can be detected again. When the marker is re-detected, there will be a sudden jump in positional coordinates. The static output of angles during the time of marker loss would reflect as poor correlation, thus attributing to the correlation coefficients between systems, though only slightly. Given a high correlation coefficient, the wide limits of agreement present in the Bland–Altman analysis indicate a difference of absolute values. In any case, the value discrepancies between MoCa and BioStamp may be attributed to sensor drift in BioStamp’s gyroscopic output as mentioned previously, which resulted in downward or upward shifts of angles over time.

The Bland–Altman analysis of the angular velocity between systems indicates complex relationships between systems and their output. The general trend is a proportional bias, indicated by the linear regression between the differences and the magnitude of the angular velocity. This indicates that the differences are largely dependent on the average. Visually, there is also an inverse proportional bias, with MoCa overestimating negative angular velocity values and underestimating positive values. The presence of both types of proportional bias indicates high-frequency noise on the behalf of MoCa, wherein for any given velocity value, it will either underestimate or overestimate. Though the sensor drift present in BioStamp’s gyroscopic output may also play a part, the noise in MoCa far outweighed the bias effects from BioStamp, as evident by the significance of the linear regression.

The linear speed was the third metric analyzed between the systems. Linear speed correlations were the lowest of the three correlations, and the linear regression present in the Bland–Altman plot indicates a systemic disparity between MoCa and BioStamp. MoCa displayed a large degree of noise, resulting in a great deal of fluctuations in its output, as evident in the low correlation coefficient and large proportional bias in the Bland–Altman analysis. Though MoCa’s tendency to lose a marker is attributed in part to the fluctuations in linear speed, it is much more likely that MoCa tends to have a large sensitivity to coordinate tracking and thus produces erratic linear speed data.

### 4.4. Speed Comparison

In addition to size categorization, another factor we analyzed was the speed of the motion. Both fast and slow paces were performed for each movement. Overall, the speed of the marker and stamps movement had little effect on the trackability of the motion detection systems. In Figure 15, the average angular displacements (a) and velocities (b) of all motions divided into fast and slow paces showed little difference. Linear speed (c) had a much higher value for the fast category, which is expected considering that the fast category is intended to have a faster rate of motion relative to the slow category of motions. 

The Pearson correlation coefficients of angular displacement, angular velocity, and linear speed between fast and slow are all relatively similar. Slow had slightly higher correlation scores for angular displacement, angular velocity, and linear speed which could be because slower data tracking lends itself to less tracking loss and thus, cleaner data.

### 4.5. System Comparison

The BioStamp is an FDA-approved device that can track three-dimensional motion and rotational movement. It utilizes a cloud-based system for data storage, which enables instantaneous data upload. MoCa is entirely video-based, capturing motion data in the 2D.

MoCa is an inexpensive and nimble system. It requires several colored stamps and a camera. The BioStamp is currently not easily accessible due to its high cost, limiting its accessibility for patients. The operating costs are almost negligible for MoCa, assuming a camera is available. This makes it readily deployable with a fairly fast setup time in terms of data collection; the subject needs to place the markers per location instruction, set up a camera, and start recording. As it is video-based, qualitative information in the form of video recordings can be reviewed instantly, allowing a researcher or clinician to make small adjustments as needed for data collection.

There exists a very comparable degree of processing required to obtain angular displacement, angular velocity, and linear speed from both MoCa and BioStamp as is; BioStamp requires the processing of acceleration and gyroscope data while MoCa requires the processing of positional data. BioStamp’s acceleration data requires normalization according to gravity to obtain accurate data. Static motions may benefit from a simple static normalization, while more dynamic movements may require the dynamic normalization as performed here. BioStamp’s angular velocity, like many gyroscopes, is affected by sensor drift and obtaining movement-accurate angular data may require additional processing [36]. MoCa on the other hand does not need to adjust for gravity, given its positional capture of motion. Similarly, sensor drift is inherently not an issue for MoCa. Positional data is recorded in pixels, and obtaining units such as meters requires a measurement of a reference point in the video, such as the subject’s arm. BioStamp does not need a reference measurement, given the meters can easily be obtained from its gyroscopic data, given in g’s.

Beyond necessary processing components, in advancing these technologies, both systems can benefit from varying degrees of noise-adjustment and smoothing. We examined MoCa’s output of angular displacement, angular velocity, and linear speed without any smoothing or filtering applied. Due to the amount of noise present, MoCa’s linear speed output can greatly benefit from a high-frequency filtering process to obtain relevant information. As seen by the similarity in the systems’ angular displacement, BioStamp benefitted from smoothing on its angular velocity, and by transitivity, its angular displacement as well.

MoCa has visual tracking capabilities that are not inherent to BioStamp. MoCa’s positional tracking provides a direct visual indicator of motion, given that it tracked accordingly. The inclusion of the accompanying video from which its data was derived from can also provide substantial qualitative information if need be. BioStamp only provides raw numeric metrics and requires significant processing to create comparable data to that of MoCa’s positional data. Therefore, deriving a visual interpretation of a BioStamp recorded motion can be challenging.

Obtaining angular displacement information from MoCa may be more complex than it is for BioStamp, depending on the motion captured. For motions where the angular displacement crosses zero degrees (or 360 degrees), additional calculations are required to account for this transition. Although this was resolved manually in the experiment, alternative methods can automate this process. Additionally, it is vital to clearly delineate the pivot marker from the other markers when calculating the corresponding angle. BioStamp angular displacement was obtained from the sensor that recorded the most motion, and thus has to be selected accordingly.

MoCa is susceptible to color interference. MoCa uses color values to identify and track its markers (HSV values specifically). If there is an object in the frame of a similar color to the marker, the system will not be able to properly track the desired markers. When the subjects’ clothing and markers were similar in color, MoCa would interpret the clothing articles as trackable markers, thus causing irreconcilable interference. Lighting variability can greatly affect the success of the marker tracking, causing color distortion that causes the tracking of incorrect positions from its respective video.

The observable advantages and disadvantages of both MoCa and BioStamp are summarized in Table 3.

### 4.6. Human Activity Recognition Implications

Human activity recognition (HAR) is a field of computational study that aims to automate the recognition of human activity via the input of a variety of data sources [41]. Relevant to this study, there are modalities of HAR systems that are either based on computer-vision or on inertial measurement unit (IMU) systems. HAR systems based on gyroscopes and accelerometers, such as the BioStamp sensor, have been proven to have substantial efficacy [42]. Pose-estimation, on the other hand, is a form of HAR that utilizes ‘keypoints’ or essential skeletal features from a video to delineate human motion. OpenPose is an open-source tool that utilizes keypoints extracted from videos in order to produce characteristics on motion [43]. The toolkit currently has over 100 keypoints to extract from a given video. Albeit HAR capabilities are possible via pose-estimation, it is typically effective with a multitude of keyframes; a model designed to be lightweight still required a minimum of 15 keypoints to achieve maximal accuracy [44]. Alternatively, a model designed for low-performing computers required up to 18 keypoints [45].

While HAR is theoretically feasible with MoCa architecture, the current limitation of tracking only three markers significantly affects its adaptability for HAR applications. BioStamp, on the other hand, may perform effectively within HAR contexts, given current implementations on gyroscope and accelerometer sensors.

### 4.7. Study Limitations

The lack of a ground truth with which to compare both systems is a limitation of the study. Though there was a definition for each trial in terms of the type of motion, the speed, and the recording circumstances, there is still bound to be variance from subject to subject and from trial to trial. Gathering the ground truth of any endpoint in this sense would be difficult, further complicated by the nuances of datapoints at the frequency at which either system captures data. Both MoCa and BioStamp have been tested in terms of statistical error for their respective angular displacements, displaying significantly minimal error [17,22]. This study’s accurate representation of real-world data highlights the practical applicability of motion analysis systems such as MoCa and BioStamp.

The subjects that participated in this study were all healthy. Testing on a more diverse population, including those with injury, will be useful to extend and confirm our findings in a more diverse population. Further studies will further define similarities and differences in the population for which these systems were developed for clinical use.

The study as performed was under ambient lighting. Lighting intensity was not controlled here. MoCa trackability may be affected by poor lighting or glare from the camera, which may impact the interpretation of the results [24]. The test mirrored real-world applications; the systems may have to account for a variety of lighting conditions given. This study did not take into account exactly how much light there was at the time of recording nor the type of lighting used. Examining lighting influence will be useful for future experimentation.

This study examined only six predefined motions. While we included a range of excursion sizes, future studies may benefit from other determining factors, be it axis of rotation or specific movements that affect MoCa tracking differently. In future studies, it will be beneficial to include a greater number and variety of motions involving more joints, limbs, mobility, etc., in order to determine on a motion-by-motion basis what the limiting factors are in tracking. 

### 4.8. Current Technology

A comparison study was conducted in 2023 where the extraction of useful body metrics possible from wearable sensors was explored across virtual reality sensors, motion capture, and computer vision [34]. Virtual reality tracking and computer vision gained accuracies of 96% and 100%, respectively, where high accuracy was attributed to the extensive initial calibration for each user equipping the gear and the implementation of neural networks involving significant processing power and high computational complexity (especially in the case of computer vision). Several issues were discussed in the use of these systems in the healthcare system. High-cost equipment and insufficient maintenance posed one issue. Therefore, in response to these concerns, motion capture methods were considered ideal. Motion capture involved fixed sensors on various parts of the body that were able to provide a body ‘model’ in three-dimensional space and analyze musculoskeletal motion. However, the fixed locations of the sensors to be able to communicate with each other in order to construct the body model limits its use in varying musculoskeletal diseases should the targeted areas of testing not have corresponding sensor locations. Additionally, this system was ‘extremely inconvenient’ when collecting more than one target point. As such, MoCa is an alternative to the need for non-fixed sensors and low-cost equipment as it functions as a similar motion capture without strict placement options. Unlike the experiments’ three-dimensional motion capture, this feature is not yet fully implemented in the data collection process, the next step to improving it to be a high-substance motion capture system.

### 4.9. Future Improvements

Future improvement of MoCa will address its current limitations. In order to bring MoCa analysis into three-dimensional space, two cameras, or a single stereoscopic camera, may be implemented in combination with three-dimensional markers. Integrating onboard verification of lighting conditions would make the system more concise and viable for real clinical use. To mitigate background color interference, markers could be redesigned to incorporate a unique identifying pattern in place of single-color recognition. Further processing to account for noisy data, a loss of marker tracking, and an automated method for incorporating reference units can all improve the seamlessness of the system, thus making it more applicable for the real world.

The current implementation of MoCa relies entirely on programming scripts that may have to be adjusted for the person and motion at hand. Future implementations should address this and incorporate a graphical user interface (GUI) to provide healthcare providers an intuitive program that is easy to use. The automation of any manual aspect of the data processing may also benefit the usability of the system by eliminating the time needed to set up the post-recording analysis. This may include aspects such as measuring body lengths of the subject for unit estimations and the accessibility to work on motions not tested before.

If MoCa is intended to be used for human activity recognition applications, it may benefit from attaining data from more markers. In its current implementation, MoCa cannot record more than three markers in a single video. The use of dozens of markers would bring the efficacy of MoCa in terms of HAR results to that of other keyframe-based HAR systems [45].

Given the difficulty of establishing a ground-truth when utilizing either system for clinical motion analysis, it may be beneficial to assess whether either system could be used for delineating categories from one another. This may be in the form of testing whether a system can differentiate a healthy control group from a different group, for example. The relative data output between subjects is one method of assessing the applicability of either MoCa or BioStamp.

### 4.10. Clinical Utility

The findings of this study hold significant implications for healthcare management, disease tracking and rehabilitation. MoCa’s simplicity and real-time capabilities position it as an attractive option, especially for telemedicine applications [46]. Patients can undergo motion analysis remotely, reducing the need for clinic visits and potentially alleviating healthcare system over-capacity burdens [47]. Its user-friendly setup and dynamic visualization also facilitates quick adjustments, enhancing data quality and consequent analysis.

MoCa is being studied in disease detection and monitoring, branching beyond simple exercises as studied herein. Pulmonology is currently examining MoCa applicability [48]. Chest conditions exemplified by abnormal chest movements and respiratory fluctuations can be tracked by MoCa sensors detecting variations in the chest rising and falling. Pulmonologists can view the chest fluctuations easily on graph displays outputted by MoCa software version 1.0.

The BioStamp’s precision tracking and FDA approval also aligns well with comprehensive clinical assessments. Its potential lies in in-depth analyses that demand accurate motion data, making it a suitable choice for specific research endeavors. However, the challenges of interpreting isolated data highlight the need for a careful consideration of the analysis objectives.

As an example, these systems may be used in monitoring motion abnormalities in patients with chronic kidney disease (CKD). MoCa and BioStamp provide quantifiable and accurate motion analysis that could be performed remotely. CKD has several motion abnormalities that may be indicators of disease status such as restless leg syndrome, tremors, and movement abnormalities arising from bodily fatigue and electrolyte abnormalities [32]. Should any of these signs be detected by MoCa or BioStamp remotely, the patient can be called in for further examination and therapy. Conducting remote motion abnormality detection tests can also accelerate and confirm a diagnosis limiting the need for in-person visits, or increasing the value of a visit via enhanced information gathering in the outpatient setting.

The effectiveness of either system is outlined per movement disorders and body elements in Table 4. The application of MoCa and BioStamp are dependent on the respective motions, given that certain movements lend themselves to poor tracking and others to excellent tracking.

## 5. Conclusions

In the realm of motion analysis for healthcare assessment, management, and rehabilitation, MoCa and BioStamp emerge as distinct yet promising tools, each bearing a set of unique advantages and limitations. This study compared and contrasted the motion tracking capabilities of each, defining their respective advantages and limitations. Our findings substantiate our hypotheses that MoCa excels in real-time dynamic two-dimensional visualization and affordability, while BioStamp effectively captures 3D and rotational motion at a higher costs. MoCa and BioStamp correlate well in terms of angular displacement, with lesser correlation in terms of linear speed and angular velocity. The distinct strengths of each system reinforce our hypothesis that MoCa and BioStamp both serve as valuable tools, albeit in different capacities, for motion analysis in rehabilitation and healthcare contexts. MoCa provides more accurate angular data without the need to account for sensor error, making it ideal for tracking range of motion at an affordable price. BioStamp on the other hand excels at providing acceleration data, making it ideal for tracking linear motion at a higher cost.

## Figures and Tables

**Table 1:** Existing movement size categories (small, medium, and large), the reps per second for both slow and fast paces use, and the movement area required in square feet per size.

Movement Size	Slow Pace (×3)	Fast Pace (×3)	Movement Area Requirement	MoCa Stamp Size
Small	1 rep/1 s	3 reps/1 s	1 sq ft	2 × 2 mm
Medium	1 rep/6 s	1 rep/2 s	5 sq ft	10 × 10 mm
Large	1 rep/6 s	1 rep/2 s	10 sq ft	10 × 10 mm

**Table 2:** Categorization of movements into movement sizes. The movement sizes (small, medium, and large) are differentiated by movement area (sq ft) required to perform the movement.

Movement Size	Movement Area Requirement	Movements
Small	1 sq ft	Finger Pinch
Medium	5 sq ft	Bicep Curl Chest Abduction/Adduction Shoulder Abduction/Adduction Shoulder Flexion/Extension
Large	1 rep/6 s	Body Lean

**Table 3:** Advantages and disadvantages of MoCa and BioStamp.

MoCa Disadvantages	MoCa Advantages	BioStamp Disadvantages	BioStamp Advantages
Longer processing period post recordings	Tracks angular displacement very well	Greater stamp quantity required for large movement sizes	Tracked consistently across all motions
Rotation in movement causes obscurity	Tracked well on both fast and slow	Suffers from gyroscopic sensor-drift	Tracked well on both fast and slow
Markers easily get obscured	Flexible marker placement	Strict marker placement	Data outputted quickly

**Table 4:** Sample applications of MoCa and BioStamp for the assessment of several movement disorders and their respective disease states. Organized based on body elements (whole body, extremity, digit, etc.) and offering commentary on the efficacy of each motion capture modality.

Body Element	Movement Disorders	Disease States	MoCa	BioStamp
	Limited ROM	Traumatic injury arthritis	Moderate: ROM testing may require tracking over larger distances	Moderate: Should be able to track well but perhaps be wary of motions in the vertical axis
Large Whole Body	Chorea	Lesions affecting the striatum Huntington disease Sydenham chorea Levodopa-induced dyskinesia	Poor: Unpredictable large motions may obscure marker placement and be poorly tracked	No issues
	Akathisia	Psychomotor disorder	Moderate: There are multiple manifestations of akathisia. May be appropriate for larger movements, but difficulty tracking smaller motions.	Moderate: Due to BioStamp’s difficulty tracking vertical motion, may have some trouble with tracking
	Gait Abnormalities	Parkinson disease Osteoarthritis Traumatic injury Stroke Cerebral palsy	Poor: Due to the unpredictability of ataxic gait, there is a high likelihood of markers on extremities becoming occluded and interfering with tracking	No issues
	Limited ROM	Traumatic injury arthritis	No issues	No issues
	Myoclonus	Uremia Creutzfeldt-Jakob disease Epilepsy syndromes	No issues	No issues
	Restless Leg Syndrome	Low iron stores Uremia Peripheral neuropathy	No issues	No issues
Medium Upper and Lower Extremities	Ballismus	Subthalamic nucleus lesion	Moderate: Subjects can present with large involuntary movements and may not be able to prevent marker occlusion	No issues
	Resting Tremor	Parkinson disease Lewy body dementia Multiple system atrophy Progressive supranuclear palsy Wilson disease Dopamine antagonists	No issues	No issues
	Postural Tremor	Essential tremor Orthostatic tremor	No issues	No issues
Small Wrist, Ankle, Hands, Feet, and Digits	Limited ROM	Traumatic injury arthritis	No issues	No issues
	Asterixis	Hepatic encephalopathy Uremic encephalopathy Hypercapnia	No issues	No issues

## Data Availability

The data that support the findings of this study are available on request from the corresponding author. The data are not publicly available due to privacy.
